# Volume–outcome relationship of liver surgery: a nationwide analysis

**DOI:** 10.1002/bjs.11586

**Published:** 2020-03-24

**Authors:** P. B. Olthof, A. K. E. Elfrink, E. Marra, E. J. T. Belt, P. B. van den Boezem, K. Bosscha, E. C. J. Consten, M. den Dulk, P. D. Gobardhan, J. Hagendoorn, T. N. T. van Heek, J. N. M. IJzermans, J. M. Klaase, K. F. D. Kuhlmann, W. K. G. Leclercq, M. S. L. Liem, E. R. Manusama, H. A. Marsman, J. S. D. Mieog, S. J. Oosterling, G. A. Patijn, W. te Riele, R.‐J. Swijnenburg, H. Torrenga, P. van Duijvendijk, M. Vermaas, N. F. M. Kok, D. J. Grünhagen, Dutch Hepato Biliary Audit Group, M. G. H. Besselink, M. T. de Boer, C. I. Buis, T. M. van Gulik, F. J. H. Hoogwater, I. Q. Molenaar, C. H. C. Dejong, C. Verhoef

**Affiliations:** ^1^ Department of Surgery Erasmus MC, Erasmus University Rotterdam the Netherlands; ^2^ Department of Surgery Cancer Centre Amsterdam, Amsterdam UMC, University of Amsterdam Amsterdam the Netherlands; ^3^ Department of Surgery Netherlands Cancer Institute Amsterdam the Netherlands; ^4^ Department of Surgery Onze Lieve Vrouwe Gasthuis Amsterdam the Netherlands; ^5^ Dutch Institute for Clinical Auditing, Scientific Bureau Leiden the Netherlands; ^6^ Department of Surgery Leiden University Medical Centre Leiden the Netherlands; ^7^ Department of Surgery University Medical Centre Groningen Groningen the Netherlands; ^8^ Department of Surgery Albert Schweitzer Hospital Dordrecht the Netherlands; ^9^ Department of Surgery Radboud University Medical Centre Nijmegen the Netherlands; ^10^ Department of Surgery Jeroen Bosch Hospital ‘s‐ Hertogenbosch the Netherlands; ^11^ Department of Surgery Meander Medical Centre Amersfoort the Netherlands; ^12^ Department of Surgery Maastricht University Medical Centre Maastricht the Netherlands; ^13^ Department of Surgery Amphia Hospital Breda the Netherlands; ^14^ Department of Surgery University Medical Centre Utrecht Utrecht the Netherlands; ^15^ Department of Surgery Gelderse Vallei Hospital Ede the Netherlands; ^16^ Department of Surgery Máxima Medisch Centrum Veldhoven the Netherlands; ^17^ Department of Surgery Medical Spectrum Twente Enschede the Netherlands; ^18^ Department of Surgery Medical Centre Leeuwardena Leeuwarden the Netherlands; ^19^ Department of Surgery Spaarne Gasthuis Haarlem the Netherlands; ^20^ Department of Surgery Isala Hospital Zwollea the Netherlands; ^21^ Department of Surgery Sint Antonius Hospital Nieuwegein the Netherlands; ^22^ Department of Surgery Deventer Hospital Deventer the Netherlands; ^23^ Department of Surgery Gelre Hospital Apeldoorn Apeldoorn the Netherlands; ^24^ Department of Surgery IJsselland Hospital Capelle aan den Ijssel the Netherlands

## Abstract

**Background:**

Evidence for an association between hospital volume and outcomes for liver surgery is abundant. The current Dutch guideline requires a minimum volume of 20 annual procedures per centre. The aim of this study was to investigate the association between hospital volume and postoperative outcomes using data from the nationwide Dutch Hepato Biliary Audit.

**Methods:**

This was a nationwide study in the Netherlands. All liver resections reported in the Dutch Hepato Biliary Audit between 2014 and 2017 were included. Annual centre volume was calculated and classified in categories of 20 procedures per year. Main outcomes were major morbidity (Clavien–Dindo grade IIIA or higher) and 30‐day or in‐hospital mortality.

**Results:**

A total of 5590 liver resections were done across 34 centres with a median annual centre volume of 35 (i.q.r. 20–69) procedures. Overall major morbidity and mortality rates were 11·2 and 2·0 per cent respectively. The mortality rate was 1·9 per cent after resection for colorectal liver metastases (CRLMs), 1·2 per cent for non‐CRLMs, 0·4 per cent for benign tumours, 4·9 per cent for hepatocellular carcinoma and 10·3 per cent for biliary tumours. Higher‐volume centres performed more major liver resections, and more resections for hepatocellular carcinoma and biliary cancer. There was no association between hospital volume and either major morbidity or mortality in multivariable analysis, after adjustment for known risk factors for adverse events.

**Conclusion:**

Hospital volume and postoperative outcomes were not associated.

## Graphical abstract

This study analysed the association between hospital volume and postoperative outcomes in the nationwide Dutch Hepato Biliary Audit. A total of 5590 resections was performed across 34 centres with a median annual centre volume of 35 (i.q.r. 20–69) procedures. No association was found between hospital and postoperative outcomes.

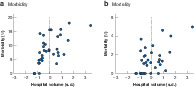

Cut‐off about right

## Abstract (es)

**Antecedentes:**

La asociación entre el volumen hospitalario y los resultados de la cirugía hepática no está clara. Según la recomendación actual de las guías holandesas se requiere un volumen mínimo de 20 procedimientos anuales por centro. El objetivo de este estudio fue analizar la asociación entre el volumen hospitalario con los resultados postoperatorios en la auditoría hepatobiliar obligatoria holandesa a nivel nacional.

**Métodos:**

Se realizó un estudio a nivel nacional en los Países Bajos. Se incluyeron todas las resecciones hepáticas registradas en la auditoría hepatobiliar holandesa entre 2014 y 2017. El volumen anual del centro se calculó y se clasificó en categorías de 20 procedimientos por año. Los objetivos principales fueron la morbilidad de mayor grado (Clavien‐Dindo grado IIIA o superior) y la mortalidad hospitalaria o la mortalidad a los 30 días.

**Resultados:**

Se realizaron un total de 5.590 resecciones en 34 centros con una mediana (rango intercuartílico) de volumen anual de 35 procedimientos (20‐69). La tasa global de morbilidad mayor fue del 11% y la mortalidad del 2%. La mortalidad fue de 1,9% después de la resección por metástasis hepáticas colorrectales (*colorectal liver metastases*, CRLM), 1,2% para no CRLM, 0,4% para tumores benignos, 4,9% para carcinoma hepatocelular, y 10,3% para tumores biliares. Los centros de mayor volumen realizaron más resecciones hepáticas mayores y más resecciones por carcinoma hepatocelular y cáncer biliar. En el análisis multivariable después de ajustar por factores de riesgo conocidos de eventos adversos, no se observó ninguna asociación entre el volumen hospitalario y la morbilidad o mortalidad mayor.

**Conclusión:**

No hubo asociación entre el volumen hospitalario y los resultados postoperatorios de la cirugía hepática en los Países Bajos.

## Introduction

In an effort to reduce morbidity and mortality after complex surgical procedures, hospital volume has become a frequent subject of debate^1^, ^2^, ^3^. Higher caseload leads to more experience for the entire surgical treatment team, which could benefit clinical outcomes. In upper gastrointestinal surgery, the volume–outcome relationship has been studied most extensively for oesophageal and pancreatic surgery^4^, ^5^, ^6^.

Mortality after oesophagectomy in low‐volume centres is at least twice that in high‐volume centres^4^. Perioperative mortality after pancreatoduodenectomy is also more than twofold lower in centres undertaking more than 40 resections compared with five procedures annually^5^, ^6^. In the Netherlands, these studies have led to a minimum annual case volume of at least 20 procedures per hospital for these operations. Without extensive evidence, this threshold has been extrapolated to hepatobiliary surgery, including liver resection.

Liver surgery, however, is more heterogeneous, with numerous different types of procedures and techniques for various indications, all with their distinct characteristics and risk factors for adverse outcomes. Procedures range from laparoscopic peripheral wedge resection to extended right liver resection with biliary reconstruction for perihilar cholangiocarcinoma. Currently there are no data to support the hospital volume standard of 20 resections annually in the Netherlands. Although improvements in perioperative care have reduced overall surgical morbidity, liver surgery is done in an ageing and increasingly co‐morbid population, and is still associated with substantial risks^7^, ^8^, ^9^, ^10^, ^11^, ^12^, ^13^, ^14^, ^15^. Adequate risk stratification across all liver surgery centres is essential to ensure optimal clinical outcomes.

This study aimed to investigate the relationship between hospital volume and postoperative outcomes after liver surgery using data from the nationwide Dutch Hepato Biliary Audit (DHBA) on all hepatobiliary resections performed in the Netherlands.

## Methods

This was a retrospective nationwide study of patients who underwent liver surgery in the Netherlands. The Netherlands is a high‐income country in Western Europe with over 17 million inhabitants. Healthcare is arranged in 121 hospitals, including eight university hospitals and one comprehensive cancer centre. In the Netherlands, requirements for treating several tumours have been defined by Stichting Oncologische Samenwerking (SONCOS), which is a national establishment for multidisciplinary oncological cooperation founded by the Dutch Societies of Surgical Oncology, Radiotherapy and Medical Oncology. Liver tumours and liver surgery are included. These guidelines are also endorsed by the government and all insurance companies. They include structural requirements such as 24/7 availability of an interventional radiologist and two skilled hepatobiliary surgeons, volume requirements for resection (at least 20 resections have to be performed annually) and mandatory participation in the audit. All hepatobiliary procedures are included in the nationwide DHBA. The present study included patients who had liver surgery for any indication between 1 January 2014 and 31 December 2017. Patients who underwent exploratory laparotomy because unresectable disease was discovered during surgery, and those who underwent extrahepatic biliary resection only were excluded. Patients with essential data missing (type of tumour, type of procedure, hospital information or operation date) were also excluded.

The study protocol was approved by the scientific committee of the DHBA. All data were handled anonymously. The need for ethical approval and individual informed consent was waived by the medical ethics committee.

### Dutch Hepato Biliary Audit

The DHBA is part of the Dutch Institute of Clinical Auditing and was initiated by the Dutch Liver Surgery Working Group. The DHBA started in 2013 and since 1 January 2014 it has been a mandatory audit for all Dutch centres performing liver surgery. The inclusion criteria are any resection for any type of liver tumour. In 2015 the registration was extended to include all procedures for biliary tumours, with the biliary tumour location specified. Before 2015, patients with biliary tumours were classified as unspecified, along with those who underwent liver resection for biliary tumours other than perihilar or intrahepatic cholangiocarcinoma. Besides direct feedback on potential errors in data entry using the online data form, voluntary external data verification was carried out. Based on this verification, data accuracy was considered adequate for all audited centres. Further details of the audit have been described elsewhere^14^.

### Hospital volume

The total number of liver resections performed during the study in each centre was recorded and the median number of procedures annually was defined as the hospital volume. In centres where liver surgery was discontinued during the study interval (all owing to an annual procedural volume below 20), only the years in which liver surgery was performed were used in the volume calculation. Extrahepatic biliary resections and exploratory laparotomies without liver resection were not included in hospital volume calculations. The annual hospital volume was classified into fewer than 20, 20–39, 40–59, 60–79 and 80 or more procedures. For multivariable analyses, the 20–39 category was used as reference because of the existing volume requirement of 20 resections annually.

### Definitions

Major liver resection was defined as resection of at least three adjacent Couinaud segments. All complications within 30 days after surgery were scored and graded according to the Clavien–Dindo classification^16^. Major morbidity was defined as the presence of a grade IIIA or higher complication. A complicated postoperative course was defined by: hospital stay exceeding 14 days, major morbidity or death. Postoperative mortality was defined as death in hospital or within 30 days after surgery; 90‐day mortality was not included in the audit.

### Statistical analysis

Categorical variables are shown as numbers with percentages, and differences between these variables were tested using χ^2^ or Fisher's exact test. Continuous variables are presented as median (i.q.r.), unless indicated otherwise, with differences tested using Mann–Whitney *U* or Kruskal–Wallis test. Univariable and multivariable logistic regression analyses were used to identify factors associated with outcomes. Variables associated with the outcome (*P* < 0·100, Wald test) in univariable analysis were included in a multivariable model. Backward selection was used in order to obtain a parsimonious model. Multivariable analyses for major morbidity and mortality were conducted on the entire cohort. The analysis for major morbidity was repeated in the subgroups of minor liver resection, major liver resection, and resection for colorectal liver metastases. Multicollinearity was assessed in all models by calculation of the variance inflation factor (VIF). A VIF of 2·5 is equivalent to an *R* ^2^ of 0·6 between two variables, and correlations with a VIF above 2·5 were considered troublesome. Sensitivity analyses were performed for every multivariable model to determine the dependency of the model outcome on specific variables. The analyses were repeated with the inclusion of additional parameters: centre volume of major liver resections (more than 10 procedures), and the year of surgery. *P* < 0·050 was considered to indicate statistical significance. All analyses were performed using R (R Foundation for Statistical Computing, Vienna, Austria).

## Results

### Liver surgery in the Netherlands

A total of 6094 procedures were undertaken. Seventy‐four extrahepatic biliary resections and 267 procedures for unresectable tumours were excluded. A further 163 procedures were excluded owing to missing essential data. Finally, 5590 procedures were included in the analyses. These procedures were done across 34 centres with a median of 35 (i.q.r. 20–69) annually. Overall, 13·7 per cent of the patients had a complicated postoperative course. The major morbidity rate was 11·2 per cent and the postoperative mortality rate was 2·0 per cent. The postoperative course was complicated after 9·2 per cent of the 4210 minor liver resections, and major morbidity and mortality rates in these patients were 7·8 and 1·2 per cent respectively. After 1380 major liver resections, 26·0 per cent of patients had a complicated postoperative course; the major morbidity rate was 20·8 per cent and the mortality rate 5·9 per cent.

**Figure 1:**
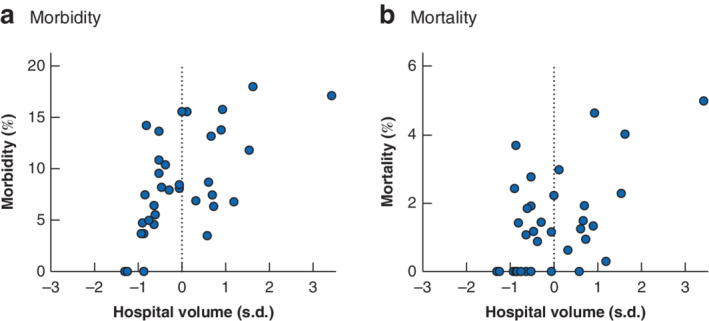
Major morbidity and mortality according to mean centre volume in the Dutch Hepato Biliary Audit between 2014 and 2017 **a** Major morbidity and **b** mortality.

An increase in the rate of adverse events was observed in higher‐volume centres in the overall cohort (*Table* 1 and *Fig*. 1). Of all 1062 resections performed in centres with fewer than 40 resections per year, 816 (76·8 per cent) were minor resections for colorectal liver metastases (CRLMs) or benign lesions, and only 21 (2·0 per cent) were major resections for hepatocellular carcinoma (HCC) or biliary tumours.

**Table 1:** Patient and treatment characteristics and outcomes according to hospital volume in the Dutch Hepato Biliary Audit between 2014 and 2017

	Hospital volume (annual no. of liver resections)					
	< 20 (*n* = 196)	20–39 (*n* = 866)	40–59 (*n* = 822)	60–79 (*n* = 1953)	≥ 80 (*n* = 1753)	*P* †
**Baseline characteristics**						
Age > 70 years	75 (38·3)	345 (39·8)	295 (35·9)	600 (30·7)	444 (25·3)	0·035
Men	117 (59·7)	503 (58·1)	462 (56·2)	1146 (58·7)	947 (54·0)	< 0·001
ASA fitness grade > III	26 (13·3)	199 (23·0)	133 (16·2)	363 (18·6)	302 (17·2)	< 0·001
BMI (kg/m^2^)*	26·9(4·4)	25·9(4·2)	26·2(4·6)	26·5(4·6)	26·1(4·5)	0·076‡
Charlson co‐morbidity index score ≥ 2	70 (35·7)	264 (30·5)	210 (25·5)	542 (27·8)	409 (23·3)	< 0·001
Preoperative liver disease	1 (0·5)	41 (4·7)	18 (2·2)	71 (3·6)	94 (5·4)	< 0·001
Previous liver surgery	23 (12·5)	149 (17·3)	122 (14·8)	272 (13·9)	206 (11·8)	0·003
Preoperative chemotherapy	16 (8·8)	180 (22·1)	150 (18·2)	295 (15·1)	483 (27·6)	< 0·001
Preoperative portal vein embolization	0 (0)	10 (3·2)	10 (1·2)	34 (1·7)	61 (3·5)	< 0·001
**Type of tumour**						< 0·001
Colorectal liver metastasis	166 (84·7)	723 (83·5)	649 (79·0)	1304 (66·8)	1004 (57·3)	
Liver metastasis from other origin	11 (5·6)	34 (3·9)	61 (7·4)	146 (7·5)	150 (8·6)	
Benign liver tumour	15 (7·7)	58 (6·7)	42 (5·1)	190 (9·7)	214 (12·2)	
Hepatocellular carcinoma	4 (2·0)	36 (4·2)	55 (6·7)	201 (10·3)	254 (14·5)	
Malignant tumour of biliary tract	0 (0)	15 (1·7)	15 (1·8)	112 (5·7)	131 (7·5)	
**Operative characteristics and outcomes**						
Major liver resection	29 (14·8)	130 (15·0)	240 (29·2)	432 (22·1)	549 (31·3)	< 0·001
Minimally invasive approach	17 (8·7)	259 (29·9)	140 (17·0)	488 (25·0)	198 (11·3)	< 0·001
Intraoperative ablation	7 (3·6)	109 (12·6)	161 (19·6)	292 (15·0)	289 (16·5)	< 0·001
Simultaneous colorectal resection	36 (18·4)	112 (12·9)	92 (11·2)	144 (7·4)	139 (7·9)	< 0·001
Simultaneous other resection	70 (35·7)	185 (21·4)	118 (14·4)	306 (15·7)	207 (11·8)	< 0·001
Biliary reconstruction	0 (0)	1 (0·1)	3 (0·4)	47 (2·4)	44 (2·5)	< 0·001
Complicated postoperative course	22 (11·2)	97 (11·2)	122 (14·8)	230 (11·8)	297 (16·9)	< 0·001
Major morbidity	16 (8·2)	73 (8·4)	90 (10·9)	197 (10·1)	249 (14·2)	< 0·001
Postoperative bile leakage	5 (2·6)	28 (3·2)	27 (3·3)	67 (3·4)	111 (6·3)	< 0·001
Postoperative haemorrhage	2 (1·0)	7 (0·8)	11 (1·3)	19 (1·0)	27 (1·5)	< 0·001
Postoperative liver failure	2 (1·0)	11 (1·3)	9 (1·1)	34 (1·7)	48 (2·7)	0·016
Death	2 (1·0)	12 (1·4)	11 (1·3)	32 (1·6)	57 (3·3)	< 0·001

Values in parentheses are percentages unless indicated otherwise;

* values are mean(s.d.).

† χ^2^ or Fisher's exact test, except

‡ Kruskal–Wallis test.

### Outcomes according to disease subgroups

*Table* 2 shows morbidity and mortality rates according to indication for liver surgery. For minor liver resections, morbidity and mortality rates were similar across different volume centres for most diagnoses.

**Table 2:** Tumour‐specific major morbidity and mortality rate after major and minor liver resections, and liver resection for biliary tumours according to hospital volume in the Dutch Hepato Biliary Audit between 2014 and 2017

	Hospital volume (annual no. of liver resections)					
	< 20	20–39	40–59	60–79	≥ 80	Overall
**Minor liver resection**						
Colorectal liver metastases						
No. of procedures	141	605	463	1057	751	3017
Morbidity (%)	7·1	7·4	7·6	6·9	9·5	9·1
Mortality (%)	0·7	0·3	0·6	0·6	1·2	1·4
Non‐colorectal liver metastases						
No. of procedures	8	32	44	129	122	335
Morbidity (%)	0	6	14	8·5	4·1	7·1
Mortality (%)	0	0	0	0	0	0·3
Benign tumour						
No. of procedures	15	55	31	154	148	403
Morbidity (%)	7	9	3	7·8	6·1	6·9
Mortality (%)	0	0	0	0·6	0	0·2
Hepatocellular carcinoma						
No. of procedures	3	31	36	136	134	340
Morbidity (%)	0	3	22	7·4	11·2	10·0
Mortality (%)	0	0	6	0	3·0	2·1
**Major liver resection**						
Colorectal liver metastases						
Number	25	118	186	247	253	829
Morbidity (%)	16	25·4	11·8	16·2	17·8	15·4
Mortality (%)	0	6·8	0·5	4·0	4·7	3·7
Non‐colorectal liver metastases						
No. of procedures	3	2	17	17	28	67
Morbidity (%)	0	50	18	24	27	24
Mortality (%)	0	0	6	6	7	5·9
Benign tumour						
No. of procedures	0	3	11	36	66	116
Morbidity (%)	–	0	36	19	23	10·2
Mortality (%)	–	0	0	3	0	0·9
Hepatocellular carcinoma						
No. of procedures	1	5	19	65	120	210
Morbidity (%)	100	0	16	20	25·0	22·4
Mortality (%)	100	0	16	8	9·2	9·5
**Liver resection for biliary tumours**						
Overall						
No. of procedures	0	15	15	112	131	273
Morbidity (%)	–	13	53	24·1	38·9	32·2
Mortality (%)	–	0	7	7·1	14·5	10·3
Perihilar						
No. of procedures	0	1	3	46	50	100
Morbidity (%)	–	0	100	33	58	47·0
Mortality (%)	–	0	0	11	18	14·0
Intrahepatic						
No. of procedures	0	5	4	44	44	97
Morbidity (%)	–	20	50	16	34	26
Mortality (%)	–	0	25	2	18	10
Extrahepatic / unspecified						
No. of procedures	0	9	8	22	37	76
Morbidity (%)	–	11	38	23	19	21
Mortality (%)	–	0	0	9	5	5

Morbidity and mortality for biliary tumours subdivided according to tumour location are shown in *Table* 2. Morbidity and mortality rates were highest for perihilar cholangiocarcinoma (47·0 and 14·0 per cent respectively).

The 550 resections for HCC over 4 years were performed across 22 centres, with 20 centres still undertaking HCC resections in 2017. The 273 resections for biliary tumours were carried out in 16 centres. Fourteen centres performed resections for perihilar and intrahepatic cholangiocarcinoma.

### Multivariable analyses

Although the odds ratio for major morbidity and mortality was significantly higher for centres undertaking at least 80 liver resections annually compared with those performing 20–39 procedures in univariable analyses, there was no hospital volume–outcome relationship in multivariable analyses for major morbidity and mortality in the overall cohort after correction for other confounding variables (*Table* 3; *Table* *S1*, supporting information). The multivariable analyses for major morbidity and mortality were repeated for minor resection only (*Table* *S2*, supporting information), major resections (*Table* *S3*, supporting information) and resections for CRLMs (*Table* *S4*, supporting information). None of these analyses demonstrated a hospital volume–outcome effect. Multicollinearity was not a problem as the VIF was below 2·5 for all variables in all models. Sensitivity analyses were undertaken for all outcomes stratified by tumour type. In addition, all logistic regression analyses were performed with addition of a variable concerning annual major resection volume (more than 10) and with addition of a variable correcting for year of surgery. These variables were not significant predictors of outcomes and did not alter the results of the multivariable models.

**Table 3:** Univariable and multivariable logistic regression analyses for factors associated with major morbidity including hospital volume in the Dutch Hepato Biliary Audit between 2014 and 2017

		Univariable analysis		Multivariable analysis	
	No. of patients	Odds ratio	*P*	Odds ratio	*P*
**Age (years)**			< 0·001		< 0·001
≤ 70	3811	1·00 (reference)		1·00 (reference)	
> 70	1759	1·22 (1·03, 1·45)		1·12 (0·93, 1·39)	
Missing*	20				
**Sex**			0·026		0·221
M	3175	1·00 (reference)		1·00 (reference)	
F	2399	0·67 (0·57, 0·80)		0·63 (0·53, 0·78)	
Missing*	16				
**ASA fitness grade**			< 0·001		< 0·001
I–II	4388	1·00 (reference)		1·00 (reference)	
≥ III	1023	1·86 (1·53, 2·25)		1·77 (1·39, 2·15)	
Missing*	179				
**Charlson co‐morbidity index score**			< 0·001		0·002
0–1	3909	1·00 (reference)		1·00 (reference)	
≥ 2	1495	1·53 (1·28, 1·82)		1·44 (1·14, 1·74)	
Missing*	186				
**BMI (per kg/m** ^**2**^ **)**		1·00 (0·98, 1·02)	0·963		
**Liver co‐morbidity**			0·029		0·998
No	5043	1·00 (reference)		1·00 (reference)	
Yes	225	1·51 (1·03, 2·15)		1·01 (0·63, 1·56)	
Missing*	322				
**Previous resection**			0·721		
No	4620	1·00 (reference)			
Yes	772	0·96 (0·74, 1·21)			
Missing*	198				
**Type of tumour**			< 0·001		< 0·001
CRLM	3846	1·00 (reference)		1·00 (reference)	
Other liver metastasis	402	1·06 (0·74, 1·48)	0·725	1·03 (0·69, 1·48)	0·889
Benign	519	1·12 (0·82, 1·50)	0·470	1·48 (1·01, 2·09)	0·044
HCC	550	1·66 (1·28, 2·14)	< 0·001	1·20 (0·92, 1·74)	0·147
Cholangiocarcinoma	273	4·58 (3·46, 6·02)	< 0·001	3·61 (2·60, 3·03)	< 0·001
**Preoperative chemotherapy**			0·367		
No	4062	1·00 (reference)			
Yes	1124	0·91 (0·73, 1·12)			
Missing*	404				
**Procedure**			0·185		
Resection	4732	1·00 (reference)			
Resection and ablation	858	0·90 (0·76, 1·05)			
Missing*	0				
**Surgical approach**			< 0·001		< 0·001
Open	4141	1·00 (reference)		1·00 (reference)	
Laparoscopic	1102	0·40 (0·30, 0·53)	< 0·001	0·57 (0·42, 0·78)	< 0·001
Conversion to open	206	0·82 (0·51, 1·26)	0·394	0·86 (0·50, 1·37)	0·532
Missing*	141				
**Major resection**			< 0·001		< 0·001
No	4107	1·00 (reference)		1·00 (reference)	
Yes	1380	3·10 (2·60, 3·68)		2·47 (2·02, 3·03)	
Missing*	103				
**Simultaneous other resection**			< 0·001		< 0·001
No	2713	1·00 (reference)		1·00 (reference)	
Yes	886	1·60 (1·28, 2·00)	< 0·001	1·57 (1·20, 2·05)	< 0·001
Missing*	1991	1·23 (1·02, 1·48)	0·031	1·14 (0·92, 1·42)	0·238
**Simultaneous colorectal resection**			0·002		< 0·001
No	4883	1·00 (reference)		1·00 (reference)	
Yes	523	1·50 (1·15, 1·92)		2·01 (1·47, 2·73)	
Missing*	184				
**Type of hospital**			< 0·001		0·914
Tertiary referral centre	3057	1·00 (reference)		1·00 (reference)	
Other	2533	0·63 (0·53, 0·75)		0·98 (0·71, 1·36)	
**Annual hospital volume**			< 0·001		0·163
20–39	866	1·00 (reference)		1·00 (reference)	
< 20	196	0·90 (0·51, 1·49)	0·692	0·82 (0·45, 1·44)	0·524
40–59	822	1·26 (0·93, 1·71)	0·143	1·16 (0·81, 1·67)	0·412
60–79	1953	1·11 (0·83, 1·49)	0·485	1·05 (0·73, 1·53)	0·783
≥ 80	1753	1·67 (1·22, 2·02)	< 0·001	1·41 (0·91, 2·19)	0·121

Values in parentheses are 95 per cent confidence intervals.

* Not included in multivariable analysis. CRLM, colorectal liver metastasis; HCC, hepatocellular carcinoma.

### Oncological margins

The association between hospital volume and oncological margin was investigated (*Table* *S5*, supporting information). Negative margins were achieved in 3251 patients with CRLMs (84·5 per cent), with more frequent positive resection margins in the higher‐volume centres. These results are likely to be explained by more advanced disease in these patients, as demonstrated by multivariable analyses that identified five or more metastases (odds ratio 1·57, 95 per cent c.i. 1·08 to 2·26) and major liver resection (odds ratio 1·37, 1·07 to 1·75) as predictors of positive margins. Centre volume was not significant in multivariable analysis. Positive margins were most frequent after resection of biliary tumours, and in particular in lower‐volume centres after resection of perihilar and intrahepatic cholangiocarcinoma (*Table* *S5*, supporting information).

## Discussion

In the Dutch setting, no association between hospital volume and morbidity or mortality was observed after liver surgery. Although there is no evidence base for the current cut‐off of 20 procedures, all data were gathered after the implementation of this current guideline and the data do not support the implementation of any lower, or need for a higher, procedure volume cut‐off. The lack of a volume–outcome relationship is likely to be explained by the existing advanced patient selection across lower‐ and higher‐volume centres in regional collaboration. There remains room for improvement in resections for HCC and biliary tumours. Reduction in the number of centres currently involved in treating HCC and biliary tumours to a few dedicated centres might help to improve outcomes for these high‐risk patients.

Few nationwide analyses reporting on morbidity and mortality after liver resection are available, and data on hospital volume are scarce. A French nationwide study^17^ that included 28 708 liver resections over 4 years reported a 90‐day mortality rate of 3·2 per cent. There was a median of four resections per centre and 20 per cent of patients had surgery in centres with a case volume below ten annual procedures. In multivariable analyses, a volume–mortality association was observed, with centres undertaking five or fewer procedures annually as a reference. The hazard ratios for mortality were similar for all hospital categories undertaking more than 11 procedures annually, demonstrating no further volume–outcome relationship above the cut‐off of 11 resections. In a large, but not nationwide, report from the USA including 11 429 patients, the overall 90‐day mortality rate was 4·9 per cent, but ranged from 7·1 per cent when the hospital volume was below ten procedures annually to 2·9 per cent when at least 50 procedures were done^18^. In another report^19^, the analyses were based on a similar data set including 2949 liver resections, with the same 4·9 per cent mortality rate. The authors concluded that only resections done in high‐volume centres, by a high‐volume surgeon, were associated with reduced mortality. The median hospital volume was low, with two procedures annually, and median surgeon volume was one. In a more recent nationwide analysis^20^, including 110 332 liver resections performed across 1136 German hospitals, the overall mortality rate was 5·8 per cent. There is no centralization in Germany. For major liver resections, mortality was lower in centres undertaking at least 44 major resections annually. Although overall centre volume was not reported, the 1136 hospitals active in liver surgery suggest that median centre volume is low; of centres performing major resections, 80 per cent undertook fewer than four major hepatectomies annually. In this German report, the mortality rate was high in specific subgroups, such as 16 per cent after extended hepatectomy and 26 per cent when combined with biliary reconstruction.

Available studies that reported on the volume–outcome relationship in liver surgery and showed lower mortality rates in high‐volume centres had low median hospital volumes of two to four procedures annually. The Dutch volume requirement of 20 liver resections annually is similar to that in several other countries^21^, ^22^. The mortality rates reported in series that used the 20‐procedure volume requirement (such as 2·0 per cent in the Netherlands and 2·1 per cent in Norway^21^) are lower than those in countries that have not implemented such a cut‐off: 3·2 per cent in France^17^, 4·9 per cent in America^18^, ^19^, 5·8 per cent in Germany^20^ and 3·1 per cent in Sweden^23^. Such direct comparisons are biased by numerous factors including different inclusion criteria, definitions and cohort characteristics.

There are several possible reasons for the lack of a volume–outcome relationship in the present cohort. First, there simply might not be a volume–outcome relationship in liver surgery after setting a threshold of 20 resections annually, and above this threshold outcomes are just as favourable in the smaller‐volume as in the higher‐volume centres. A more likely explanation could be the higher median hospital volume of 35 procedures than in other studies, and the advanced risk stratification already performed across many Dutch regional collaborations. This is demonstrated by the predominance of minor liver resections for CRLMs in the lowest‐volume centres, compared with increasing numbers of major liver resections for other indications in the higher‐volume centres. The data also indicate more advanced metastatic disease in higher‐volume centres, and that the majority of HCC and biliary cancers are treated in the largest centres.

Although the mortality rate of 2·0 per cent appears to be in the lower range of rates reported in literature, there are several areas for improvement. The mortality rate after major liver resection was 5·9 per cent overall and 3·7 per cent for CRLMs. These rates have led to the start of a detailed analysis of these fatalities, including failure‐to‐rescue parameters. This project aims to further improve stratification of patients across centres and reduce overall the mortality rate to below 1 per cent. The current Dutch guideline encourages referral of patients with HCC and biliary cancers to experienced centres; however, in 2017, 20 and 16 centres undertook surgery on HCC and biliary tumours respectively. The overall mortality rates of 4·9 per cent after HCC resection and up to 9·5 per cent after major resection are higher than rates in the largest international series from expert centres (0·8–2·9 per cent)^24^, ^25^, ^26^. For biliary tumours, the overall mortality rate was 10·3 per cent, with the highest rate for perihilar cholangiocarcinoma (14·0 per cent). Although this is consistent with an in‐hospital mortality rate of 13 per cent in a meta‐analysis^27^ of Western series, there is significant room for improvement considering that the mortality rate after perihilar cholangiocarcinoma resections in Asian centres is usually below 4 per cent^27^. In addition, margins were positive after biliary cancer resection in over one‐third of all patients. Considering these rates, the treatment of this relatively small group across 16–20 centres is unlikely to optimize the outcomes, and further centralization of these resections to a handful of dedicated centres might help to reduce adverse outcomes.

This study had several limitations. It is possible that the lack of a volume–outcome relationship in the present analysis was the result of one or more risk‐stratifying variables that were not included in the nationwide audit data set. These variables could include advanced data on (hepatic) co‐morbidity, or variables in the patient evaluation such as the assessment of remnant liver volume and function. As in other national audits, there are some missing data for non‐mandatory parameters^28^, ^29^. Furthermore, to ensure complete anonymity in the analysis from a small country such as the Netherlands, no distinction was made between hospitals' teaching status, which is a factor known to have a potential influence on outcomes^30^. Although most perioperative deaths are likely captured by the 30‐day or in‐hospital mortality variable, 90‐day mortality is likely more representative of true mortality, but is not included in the audit and was not therefore not available for the present study^31^. As the DHBA was designed for analysis of perioperative outcomes, no information regarding long‐term outcomes is collected and therefore these could not be included in the analyses.

## Collaborators

Members of the Dutch Hepato Biliary Audit Group who collaborated in this study: M. G. H. Besselink (Cancer Centre Amsterdam, Amsterdam UMC, University of Amsterdam, Amsterdam); M. T. de Boer (University Medical Centre Groningen, Groningen); C. I. Buis (University Medical Centre Groningen, Groningen); T. M. van Gulik (Cancer Centre Amsterdam, Amsterdam UMC, University of Amsterdam, Amsterdam); F. J. H. Hoogwater (University Medical Centre Groningen, Groningen); I. Q. Molenaar (University Medical Center Utrecht, Utrecht); C. H. C. Dejong (Maastricht University Medical Centre, Maastricht); C. Verhoef (Erasmus MC, Erasmus University, Rotterdam).

## Supporting information


**Table S1** Univariable and multivariable logistic regression model of patient, tumor, surgical and volume factors associated with mortality in the Dutch Hepato Billiary Audit between 2014 and 2017
**Table S2** Minor liver resections: univariable and multivariable logistic regression model of patient, tumor, surgical and volume factors associated with major morbidity (CD 3 or higher) in the Dutch Hepato Billiary Audit between 2014 and 2017
**Table S3** Major liver resections: univariable and multivariable logistic regression model of patient, tumor, surgical and volume factors associated with major morbidity (CD 3 or higher) in the Dutch Hepato Billiary Audit between 2014 and 2017
**Table S4** Colorectal liver resections: univariable and multivariable logistic regression model of patient, tumor, surgical and volume factors associated with major morbidity (CD 3 or higher) in the Dutch Hepato Billiary Audit between 2014 and 2017
**Table S5a** Negative resections margins according to tumor type and hospital volume in the Dutch Hepato Billiary Audit between 2014 and 2017
**Table S5b** Negative resections margins of biliary tumor resections according to hospital volume in the Dutch Hepato Billiary Audit between 2014 and 2017
