# Supplementary material for: Volume–outcome relationship of liver surgery: a nationwide analysis
Source: Br J Surg. 2020 Mar 24;107(7):917–26. doi: 10.1002/bjs.11586 (PMC7384098; doi:10.1002/bjs.11586)
Supplement: Supplementary file 1 — Table S1 Univariable and multivariable logistic regression model of patient, tumor, surgical and volume factors associated with mortality in the Dutch Hepato Billiary Audit between 2014 and 2017 Table S2 Minor liver resections: univariable and multivariable logistic regression model of patient, tumor, surgical and volume factors associated with major morbidity (CD 3 or higher) in the Dutch Hepato Billiary Audit between 2014 and 2017 Table S3 Major liver resections: univariable and multivariable logistic regression model of patient, tumor, surgical and volume factors associated with major morbidity (CD 3 or higher) in the Dutch Hepato Billiary Audit between 2014 and 2017 Table S4 Colorectal liver resections: univariable and multivariable logistic regression model of patient, tumor, surgical and volume factors associated with major morbidity (CD 3 or higher) in the Dutch Hepato Billiary Audit between 2014 and 2017 Table S5a Negative resections margins according to tumor type and hospital volume in the Dutch Hepato Billiary Audit between 2014 and 2017 Table S5b Negative resections margins of biliary tumor resections according to hospital volume in the Dutch Hepato Billiary Audit between 2014 and 2017 [file BJS-107-917-s001.docx]

**Volume–outcome relationship of liver surgery: a nationwide analysis**

P. B. Olthof, A. K. E. Elfrink, E. Marra, E. J. T. Belt, P. B. van den Boezem, K. Bosscha, E. C. J. Consten, M. den Dulk, P. D. Gobardhan, J. Hagendoorn, T. N. T. van Heek, J. N. M. IJzermans, J. M. Klaase, K. F. D. Kuhlmann, W. K. G. Leclercq, M. S. L. Liem, E. R. Manusama, H. A. Marsman, J. S. D. Mieog, S. J. Oosterling, G. A. Patijn, W. te Riele, R.-J. Swijnenburg, H. Torrenga, P. van Duijvendijk, M. Vermaas, N. F. M. Kok and D. J. Grünhagen, on behalf of the Dutch Hepato Biliary Audit Group

**Table S1 Univariable and multivariable logistic regression model of patient, tumor, surgical and volume factors associated with mortality in the Dutch Hepato Billiary Audit between 2014 and 2017**

|  |  | Univariable | | |  | | |
| --- | --- | --- | --- | --- | --- | --- | --- |
| Factor | N | OR | CI (95%) | P-value | OR | CI (95%) | P-value |
| Sex |  |  |  | **0.007** |  |  | 0.362 |
| Male | 3175 | 1 |  |  | 1 |  |  |
| Female | 2399 | 0.58 | 0.38 – 0.86 |  | 0.81 | 0.52 – 1.26 |  |
| Missing* | 16 |  |  |  |  |  |  |
| Age |  |  |  | **<0.001** |  |  | **0.006** |
| <70 | 3811 | 1 |  |  | 1 |  |  |
| >70 | 1759 | 1.88 | 1.29 – 2.73 |  | 1.79 | 1.17 – 2.74 |  |
| Missing* | 20 |  |  |  |  |  |  |
| ASA Score |  |  |  | **<0.001** |  |  | **<0.001** |
| 1 / 2 | 4388 | 1 |  |  | 1 |  |  |
| 3+ | 1023 | 3.45 | 2.36 – 5.03 |  | 2.79 | 1.81 – 4.28 |  |
| Missing* | 179 |  |  |  |  |  |  |
| Charlson comorbidity index |  |  |  | **<0.001** |  |  | 0.288 |
| 1 / 2 | 3909 | 1 |  |  | 1 |  |  |
| 3 | 1495 | 2.12 | 1.45 – 3.09 |  | 1.28 | 0.81 – 2.01 |  |
| Missing* | 186 |  |  |  |  |  |  |
| Body Mass Index |  | 1.03 | 0.99 – 1.07 | 0.203 |  |  |  |
| Comorbidity liver |  |  |  | **<0.001** |  |  | **0.022** |
| No | 5043 | 1 |  |  | 1 |  |  |
| Yes | 225 | 3.83 | 2.10 – 6.52 |  | 2.43 | 1.12 – 5.15 |  |
| Missing* | 322 |  |  |  |  |  |  |
| Previous resection |  |  |  | 0.599 |  |  |  |
| No | 4620 | 1 |  |  |  |  |  |
| Yes | 772 | 1.15 | 0.67 – 1.86 |  |  |  |  |
| Missing* | 198 |  |  |  |  |  |  |
| Type of tumor |  |  |  | **<0.001** |  |  | **<0.001** |
| CRLM | 3846 | 1 |  |  | 1 |  |  |
| Other liver metastasis | 402 | 0.94 | 0.33 – 2.16 | 0.904 | 1.14 | 0.39 – 2.71 | 0.781 |
| Benign | 519 | 0.28 | 0.05 – 0.90 | 0.078 | 0.19 | 0.01 – 1.02 | 0.106 |
| HCC | 550 | 3.80 | 2.33 – 6.04 | **<0.001** | 1.45 | 0.77 – 2.66 | 0.237 |
| Cholangiocarcinoma | 273 | 8.17 | 5.01 – 13.1 | **<0.001** | 3.93 | 2.15 – 7.08 | **<0.001** |
| Missing* |  |  |  |  |  |  |  |
| Preoperatieve chemotheraphy |  |  |  | 0.403 |  |  |  |
| No | 4062 | 1 |  |  |  |  |  |
| Yes | 1124 | 0.82 | 0.49 – 1.29 |  |  |  |  |
| Missing* | 404 |  |  |  |  |  |  |
| Procedure |  |  |  | **0.008** |  |  |  |
| Resection | 4732 | 1 |  |  |  |  |  |
| Resection and ablation | 858 | 0.35 | 0.15 – 0.71 |  |  |  |  |
| Major resection |  |  |  | **<0.001** |  |  | **<0.001** |
| No | 4119 | 1 |  |  | 1 |  |  |
| Yes | 1380 | 8.12 | 5.40 – 12.5 |  | 6.21 | 3.86 – 10.2 |  |
| Missing* | 91 |  |  |  |  |  |  |
| Surgical approach |  |  |  | **<0.001** |  |  | 0.261 |
| Open | 4141 | 1 |  |  | 1 |  |  |
| Laparoscopic | 1102 | 0.25 | 0.10 – 0.50 | **<0.001** | 0.60 | 0.24 – 1.29 | 0.231 |
| Conversion | 206 | 0.19 | 0.01 – 0.86 | **0.015** | 0.30 | 0.02 – 1.43 | 0.243 |
| Missing* | 141 |  |  |  |  |  |  |
| Simultaneous other resection |  |  |  | 0.941 |  |  |  |
| No | 2713 | 1 |  |  |  |  |  |
| Yes | 886 | 0.98 | 0.55 – 1.66 | 0.944 |  |  |  |
| Missing | 1991 | 1.09 | 0.73 – 1.64 | 0.650 |  |  |  |
| Simultaneous colorectal resection |  |  |  | 0.349 |  |  |  |
| No | 4883 | 1 |  |  |  |  |  |
| Yes | 523 | 0.71 | 0.32 – 1.37 |  |  |  |  |
| Type of hospital |  |  |  | **<0.001** |  |  | 0.929 |
| Tertiary referral center | 3057 | 1 |  |  | 1 |  |  |
| Other hospitals | 2533 | 0.41 | 0.27 – 0.62 |  | 0.96 | 0.41 – 2.17 |  |
| **Hospital volume per year** |  |  |  | **<0.001** |  |  | 0.334 |
| 20-39 | 835 | 1 |  |  | 1 |  |  |
| <20 | 227 | 1.00 | 0.23 – 3.24 | 0.996 | 1.09 | 0.24 – 3.70 | 0.901 |
| 40-59 | 822 | 1.03 | 0.44 – 2.42 | 0.957 | 0.63 | 0.25 – 1.57 | 0.323 |
| 60-79 | 1953 | 1.34 | 0.69 – 2.79 | 0.414 | 0.80 | 0.31 – 2.03 | 0.628 |
| >80 | 1753 | 2.52 | 1.37 – 5.09 | 0.007 | 1.27 | 0.45 – 3.66 | 0.649 |

**Table S2 Minor liver resections: univariable and multivariable logistic regression model of patient, tumor, surgical and volume factors associated with major morbidity (CD 3 or higher) in the Dutch Hepato Billiary Audit between 2014 and 2017**

|  |  | Univariable | | | Multivariable | | |
| --- | --- | --- | --- | --- | --- | --- | --- |
| Factor | N | OR | CI (95%) | P-value | OR | CI (95%) | P-value |
| Sex |  |  |  | **<0.001** |  |  | **0.006** |
| Male | 2332 | 1 |  |  | 1 |  |  |
| Female | 1760 | 0.65 | 0.51 – 0.82 |  | 0.70 | 0.53 – 0.90 |  |
| Missing* | 15 |  |  |  |  |  |  |
| Age |  |  |  | 0.104 |  |  | 0.967 |
| <70 | 2774 | 1 |  |  | 1 |  |  |
| >70 | 1317 | 1.22 | 0.96 – 1.55 |  | 0.96 | 0.76 – 1.29 |  |
| Missing* | 16 |  |  |  |  |  |  |
| ASA Score |  |  |  | **<0.001** |  |  | **<0.001** |
| 1 / 2 | 3191 | 1 |  |  | 1 |  |  |
| 3+ | 749 | 2.12 | 1.64 – 2.73 |  | 1.94 | 1.46 – 2.55 |  |
| Missing* | 167 |  |  |  |  |  |  |
| Charlson comorbidity index |  |  |  | **<0.001** |  |  | **0.007** |
| 1 / 2 | 2844 | 1 |  |  | 1 |  |  |
| 3 | 1100 | 1.58 | 1.24 – 2.01 |  | 1.52 | 1.10 – 1.88 |  |
| Missing* | 163 |  |  |  |  |  |  |
| Body Mass Index |  | 1.00 | 0.97 – 1.02 | 0.721 |  |  |  |
| Comorbidity liver |  |  |  | 0.532 |  |  |  |
| No | 3672 | 1 |  |  |  |  |  |
| Yes | 163 | 1.19 | 0.66 – 1.99 |  |  |  |  |
| Missing* | 272 |  |  |  |  |  |  |
| Previous resection |  |  |  | 0.898 |  |  |  |
| No | 3342 | 1 |  |  |  |  |  |
| Yes | 596 | 0.98 | 0.70 – 1.34 |  |  |  |  |
| Missing* | 169 |  |  |  |  |  |  |
| Type of tumor |  |  |  | 0.148 |  |  | 0.587 |
| CRLM | 3017 | 1 |  |  | 1 |  |  |
| Other liver metastasis | 335 | 0.92 | 0.58 – 1.39 | 0.700 | 0.82 | 0.50 – 1.29 | 0.422 |
| Benign | 403 | 0.89 | 0.58 – 1.31 | 0.567 | 1.18 | 0.72 – 1.90 | 0.472 |
| HCC | 340 | 1.32 | 0.89 – 1.90 | 0.149 | 1.17 | 0.79 – 1.83 | 0.358 |
| Cholangiocarcinoma | 12 | 1.08 | 0.06 – 5.59 | 0.943 | 1.57 | 0.38 – 5.13 | 0.429 |
| Preoperatieve chemotheraphy |  |  |  | 0.981 |  |  |  |
| No | 3003 | 1 |  |  |  |  |  |
| Yes | 760 | 1.00 | 0.74 – 1.33 |  |  |  |  |
| Missing* | 344 |  |  |  |  |  |  |
| Procedure |  |  |  | 0.255 |  |  |  |
| Resection | 3361 | 1 |  |  |  |  |  |
| Resection and ablation | 746 | 1.18 | 0.88 – 1.56 |  |  |  |  |
| Surgical approach |  |  |  | **<0.001** |  |  | **0.011** |
| Open | 2865 | 1 |  |  | 1 |  |  |
| Laparoscopic | 1021 | 0.58 | 0.42 – 0.78 | <0.001 | 0.62 | 0.44 – 0.86 | **0.005** |
| Conversion | 172 | 1.31 | 0.78 – 2.10 | 0.282 | 1.09 | 0.62 – 1.82 | 0.743 |
| Missing* | 49 |  |  |  |  |  |  |
| Simultaneous other resection |  |  |  | **<0.001** |  |  | **0.001** |
| No | 2010 | 1 |  |  | 1 |  |  |
| Yes | 705 | 2.33 | 1.74 – 3.11 | **<0.001** | 2.05 | 1.46 – 2.87 | **<0.001** |
| Missing | 1404 | 1.51 | 1.16 – 1.97 | **0.002** | 1.39 | 1.04 – 1.86 | **0.029** |
| Simultaneous colorectal resection |  |  |  | **<0.001** |  |  | **<0.001** |
| No | 3488 | 1 |  |  | 1 |  |  |
| Yes | 467 | 2.19 | 1.63 – 2.91 |  | 1.70 | 1.20 – 2.40 |  |
| Missing* | 164 |  |  |  |  |  |  |
| Type of hospital |  |  |  | 0.079 |  |  | 0.569 |
| Tertiary referral center | 2115 | 1 |  |  | 1 |  |  |
| Other hospitals | 2004 | 0.81 | 0.65 – 1.02 |  | 0.89 | 0.60 – 1.33 |  |
| **Hospital volume per year** |  |  |  | 0.345 |  |  | 0.321 |
| 20-39 | 695 | 1 |  |  | 1 |  |  |
| <20 | 197 | 0.80 | 0.40 – 1.48 | 0.514 | 0.75 | 0.37 – 1.42 | 0.402 |
| 40-59 | 575 | 1.20 | 0.80 – 1.80 | 0.374 | 1.31 | 0.86 – 2.01 | 0.196 |
| 60-79 | 1489 | 0.95 | 0.67 – 1.35 | 0.760 | 0.95 | 0.61 – 1.47 | 0.827 |
| >80 | 1163 | 1.19 | 0.84 – 1.69 | 0.332 | 1.14 | 0.70 – 1.96 | 0.623 |

**Table S3 Major liver resections: univariable and multivariable logistic regression model of patient, tumor, surgical and volume factors associated with major morbidity (CD 3 or higher) in the Dutch Hepato Billiary Audit between 2014 and 2017**

|  |  | Univariable | | | Multivariable | | |
| --- | --- | --- | --- | --- | --- | --- | --- |
| Factor | N | OR | CI (95%) | P-value | OR | CI (95%) | P-value |
| Sex |  |  |  | **0.005** |  |  | **<0.001** |
| Male | 802 | 1 |  |  | 1 |  |  |
| Female | 577 | 0.68 | 0.51 – 0.89 |  | 0.56 | 0.40 – 0.76 |  |
| Missing* | 1 |  |  |  |  |  |  |
| Age |  |  |  | **0.020** |  |  | **0.041** |
| <70 | 967 | 1 |  |  | 1 |  |  |
| >70 | 409 | 1.39 | 1.05 – 1.82 |  | 1.39 | 1.01 – 1.91 |  |
| Missing* | 4 |  |  |  |  |  |  |
| ASA Score |  |  |  | **0.001** |  |  | 0.058 |
| 1 / 2 | 1118 | 1 |  |  | 1 |  |  |
| 3+ | 250 | 1.65 | 1.20 – 2.25 |  | 1.41 | 0.98 – 2.00 |  |
| Missing* | 12 |  |  |  |  |  |  |
| Charlson comorbidity index |  |  |  | **0.009** |  |  | 0.136 |
| 1 / 2 | 997 | 1 |  |  | 1 |  |  |
| 3 | 362 | 1.46 | 1.10 – 1.93 |  | 1.30 | 0.92 – 1.83 |  |
| Missing* | 21 |  |  |  |  |  |  |
| Body Mass Index |  | 1.03 | 1.00 – 1.06 | 0.069 | 1.03 | 1.00 – 1.07 | 0.052 |
| Comorbidity liver |  |  |  | **0.023** |  |  | 0.352 |
| No | 1276 | 1 |  |  | 1 |  |  |
| Yes | 57 | 1.94 | 1.08 – 3.37 |  | 1.40 | 0.68 – 2.80 |  |
| Missing* | 47 |  |  |  |  |  |  |
| Previous resection |  |  |  | 0.647 |  |  |  |
| No | 1181 | 1 |  |  |  |  |  |
| Yes | 172 | 1.09 | 0.74 – 1.60 |  |  |  |  |
| Missing* | 37 |  |  |  |  |  |  |
| Type of tumor |  |  |  | **0.021** |  |  | **0.002** |
| CRLM | 829 | 1 |  |  | 1 |  |  |
| Other liver metastasis | 67 | 1.72 | 0.92 – 3.04 | 0.072 | 1.42 | 0.69 – 1.88 | 0.319 |
| Benign | 116 | 1.58 | 0.97 – 2.51 | 0.058 | 2.00 | 1.09 – 3.58 | 0.023 |
| HCC | 210 | 1.60 | 1.08 – 2.29 | 0.021 | 1.14 | 0.69 – 1.88 | 0.594 |
| Cholangiocarcinoma | 158 | 4.36 | 3.02 – 6.28 | <0.001 | 4.00 | 2.50 – 6.47 | **<0.001** |
| Preoperative chemotherapy |  |  |  | **0.012** |  |  | 0.653 |
| No | 956 | 1 |  |  | 1 |  |  |
| Yes | 364 | 0.67 | 0.48 – 0.91 |  | 1.10 | 0.73 – 1.63 |  |
| Missing* | 60 |  |  |  |  |  |  |
| Procedure |  |  |  | **0.047** |  |  | 0.282 |
| Resection | 1268 | 1 |  |  | 1 |  |  |
| Resection and ablation | 112 | 0.57 | 0.31 – 0.96 |  | 0.72 | 0.37 – 1.28 |  |
| Surgical approach |  |  |  | **0.016** |  |  | **0.046** |
| Open | 1260 | 1 |  |  | 1 |  |  |
| Laparoscopic | 80 | 0.40 | 0.18 – 0.79 | 0.015 | 0.37 | 0.14 – 0.83 | **0.027** |
| Conversion | 34 | 0.35 | 0.08 – 0.98 | 0.083 | 0.42 | 0.10 – 1.25 | 0.170 |
| Missing* | 6 |  |  |  |  |  |  |
| Simultaneous other resection |  |  |  | 0.573 |  |  |  |
| No | 700 | 1 |  |  |  |  |  |
| Yes | 180 | 1.12 | 0.75 – 1.65 | 0.574 |  |  |  |
| Missing | 500 | 0.95 | 0.71 – 1.26 | 0.717 |  |  |  |
| Simultaneous colorectal resection |  |  |  | 0.442 |  |  |  |
| No | 1304 | 1 |  |  |  |  |  |
| Yes | 56 | 1.28 | 0.66 – 2.31 |  |  |  |  |
| Missing* | 20 |  |  |  |  |  |  |
| Type of hospital |  |  |  | **0.009** |  |  | 0.631 |
| Tertiary referral center | 870 | 1 |  |  | 1 |  |  |
| Other hospitals | 510 | 0.55 | 0.41 – 0.73 |  | 0.99 | 0.66 – 1.97 |  |
| **Hospital volume per year** |  |  |  | **0.001** |  |  | 0.267 |
| 20-39 | 129 | 1 |  |  | 1 |  |  |
| <20 | 30 | 1.16 | 0.36 – 3.21 | 0.791 | 0.96 | 0.25 – 3.05 | 0.948 |
| 40-59 | 240 | 1.02 | 0.57 – 1.90 | 0.938 | 0.97 | 0.50 – 1.94 | 0.918 |
| 60-79 | 432 | 1.40 | 0.83 – 2.46 | 0.233 | 1.17 | 0.58 – 2.41 | 0.572 |
| >80 | 549 | 2.04 | 1.23 – 3.53 | **0.007** | 1.73 | 0.80 – 3.86 | 0.166 |

**Table S4 Colorectal liver resections: univariable and multivariable logistic regression model of patient, tumor, surgical and volume factors associated with major morbidity (CD 3 or higher) in the Dutch Hepato Billiary Audit between 2014 and 2017**

|  |  | Univariable | | | Multivariable | | |
| --- | --- | --- | --- | --- | --- | --- | --- |
| Factor | N | OR | CI (95%) | P-value | OR | CI (95%) | P-value |
| Sex |  |  |  | **0.003** |  |  | **0.004** |
| Male | 2393 | 1 |  |  | 1 |  |  |
| Female | 1439 | 0.70 | 0.55 – 0.89 |  | 0.70 | 0.54 – 0.89 |  |
| Missing* | 14 |  |  |  |  |  |  |
| Age |  |  |  | 0.138 |  |  |  |
| <70 | 2467 | 1 |  |  |  |  |  |
| >70 | 1368 | 1.18 | 0.95 – 1.48 |  |  |  |  |
| Missing* | 11 |  |  |  |  |  | **<0.001** |
| ASA Score |  |  |  | **<0.001** |  |  |  |
| 1 / 2 | 3047 | 1 |  |  | 1 |  |  |
| 3+ | 660 | 1.85 | 1.43 – 2.37 |  | 1.97 | 1.43 – 2.44 |  |
| Missing* | 139 |  |  |  |  |  |  |
| Charlson comorbidity index |  |  |  | **0.028** |  |  | 0.087 |
| 1 / 2 | 2767 | 1 |  |  | 1 |  |  |
| 3 | 949 | 1.31 | 1.03 – 1.65 |  | 1.26 | 0.96 – 1.63 |  |
| Missing* | 130 |  |  |  |  |  |  |
| Body Mass Index |  | 1.00 | 0.98 – 1.03 | 0.825 |  |  |  |
| Comorbidity liver |  |  |  | 0.966 |  |  |  |
| No | 3569 | 1 |  |  |  |  |  |
| Yes | 42 | 0.98 | 0.29 – 2.45 |  |  |  |  |
| Missing* | 235 |  |  |  |  |  |  |
| Previous resection |  |  |  | 0.446 |  |  |  |
| No | 3061 | 1 |  |  |  |  |  |
| Yes | 660 | 1.11 | 0.84 – 1.46 |  |  |  |  |
| Missing* | 125 |  |  |  |  |  |  |
| Preoperative chemotherapy |  |  |  | 0.130 |  |  |  |
| No | 2439 | 1 |  |  |  |  |  |
| Yes | 1051 | 1.20 | 0.94 – 1.52 |  |  |  |  |
| Missing* | 356 |  |  |  |  |  |  |
| Procedure |  |  |  | 0.879 |  |  |  |
| Resection | 3061 | 1 |  |  |  |  |  |
| Resection and ablation | 785 | 1.02 | 0.78 – 1.33 |  |  |  |  |
| Missing* | 0 |  |  |  |  |  |  |
| Major resection |  |  |  | **<0.001** |  |  | **<0.001** |
| No | 3017 | 1 |  |  | 1 |  |  |
| Yes | 829 | 2.17 | 1.72 – 2.73 |  | 2.20 | 1.71 – 2.82 |  |
| Missing |  |  |  |  |  |  |  |
| Surgical approach |  |  |  | **0.001** |  |  | **0.047** |
| Open | 2977 | 1 |  |  | 1 |  |  |
| Laparoscopic | 685 | 0.56 | 0.39 – 0.77 | **<0.001** | 0.65 | 0.45 – 0.92 | **0.021** |
| Conversion | 149 | 0.91 | 0.50 – 1.54 | 0.741 | 0.74 | 0.38 – 1.32 | 0.357 |
| Missing* | 35 |  |  |  |  |  |  |
| Simultaneous other resection |  |  |  | **<0.001** |  |  | **0.011** |
| No | 1927 | 1 |  |  | 1 |  |  |
| Yes | 638 | 2.10 | 1.59 – 2.77 | **<0.001** | 1.78 | 1.28 – 2.46 | **<0.001** |
| Missing | 1281 | 1.37 | 1.07 – 1.76 | **0.013** | 1.28 | 0.97 – 1.68 | 0.080 |
| Simultaneous colorectal resection |  |  |  | **<0.001** |  |  | **<0.001** |
| No | 3226 | 1 |  |  | 1 |  |  |
| Yes | 461 | 2.03 | 1.53 – 2.66 |  | 1.96 | 1.40 – 2.72 |  |
| Missing* | 159 |  |  |  |  |  |  |
| Type of hospital |  |  |  | **0.081** |  |  | 0.544 |
| Tertiary referral center | 1724 | 1 |  |  | 1 |  |  |
| Other hospitals | 2122 | 0.82 | 0.66 – 1.02 |  | 1.00 | 0.59 – 1.68 |  |
| Hospital volume per year |  |  |  | 0.118 |  |  | 0.407 |
| 20-39 | 694 | 1 |  |  | 1 |  |  |
| <20 | 195 | 0.86 | 0.46 – 1.52 | 0.621 | 0.78 | 0.40 – 1.42 | 0.431 |
| 40-59 | 649 | 1.00 | 0.68 – 1.46 | 0.996 | 1.00 | 0.68 – 1.49 | 0.976 |
| 60-79 | 1304 | 0.98 | 0.71 – 1.37 | 0.926 | 1.16 | 0.78 – 1.73 | 0.448 |
| >80 | 1004 | 1.36 | 0.98 – 1.89 | 0.071 | 1.51 | 0.89 – 2.57 | 0.129 |

**Table S5a Negative resections margins according to tumor type and hospital volume in the Dutch Hepato Billiary Audit between 2014 and 2017**

|  | | **< 20** | **20-39** | **40-59** | **60-79** | **≥ 80** | **All** |
| --- | --- | --- | --- | --- | --- | --- | --- |
| ***Colorectal liver metastases*** | *Number*  *R0 resection*  *Missing* | 195  176 (90.3)  1 (0.5) | 694  630 (90.8)  4 (0.6) | 649  594 (91.5)  3 (0.5) | 1304  1004 (77.0)  128 (9.8) | 1004  847 (84.4)  8 (0.8) | 3846  3251 (84.5)  144 (3.7) |
| ***Non colorectal liver metastases*** | *Number*  *R0 resection*  *Missing* | 12  11 (91.7)  0 (-) | 33  28 (84.8)  1 (3.0) | 61  56 (91.8)  0 (-) | 146  103 (70.5)  24 (16.4) | 150  132 (88.0)  0 (-) | 402  330 (82.1)  25 (6.2) |
| ***HCC*** | *Number*  *R0 resection*  *Missing* | 5  5 (100)  0 (-) | 35  34 (97.1)  0 (-) | 55  50 (90.9)  0 (-) | 201  159 (79.1)  21 (10.4) | 254  222 (87.4)  7 (2.8) | 550  470 (85.5)  28 (5.1) |

**Table S5b Negative resections margins of biliary tumor resections according to hospital volume in the Dutch Hepato Billiary Audit between 2014 and 2017**

|  | | **< 20** | **20-39** | **40-59** | **60-79** | **≥ 80** | **All** |
| --- | --- | --- | --- | --- | --- | --- | --- |
| ***Biliary*** | *Number*  *R0 resection*  *Missing* | 0  -  - | 15  3 (20.0)  11 (73.3) | 15  6 (40.0)  5 (33.3) | 112  65 (58.0)  30 (26.8) | 131  73 (55.7)  41 (31.3) | 273  147 (53.8)  87 (31.7) |
| ***Perihilar*** | *Number*  *R0 resection*  *Missing* | 0  -  - | 1  0 (-)  1 (100) | 3  1 (33.3)  0 (-) | 46  31 (67.4)  4 (8.7) | 50  35 (70.0)  1 (2.0) | 100  67 (67.0)  6 (6.0) |
| ***Intrahepatic*** | *Number*  *R0 resection*  *Missing* | 0  -  - | 5  3 (60.0)  2 (40.0) | 4  2 (50.0)  1 (25.0) | 44  24 (54.5)  15 (34.1) | 44  31 (70.5)  11 (25.0) | 97  60 (61.9)  29 (29.9) |
| ***Unspecified*** | *Number*  *R0 resection*  *Missing* | 0  -  - | 9  0 (-)  8 (88.9) | 8  3 (37.5)  4 (50.0) | 22  10 (45.5)  11 (50.0) | 37  7 (18.9)  29 (78.4) | 76  20 (26.3)  52 (68.4) |
